# Accurate brain tumor detection using deep convolutional neural network

**DOI:** 10.1016/j.csbj.2022.08.039

**Published:** 2022-08-27

**Authors:** Md. Saikat Islam Khan, Anichur Rahman, Tanoy Debnath, Md. Razaul Karim, Mostofa Kamal Nasir, Shahab S. Band, Amir Mosavi, Iman Dehzangi

**Affiliations:** a Department of CSE, Mawlana Bhashani Science and Technology University, Tangail, Bangladesh; b Department of CSE, National Institute of Textile Engineering and Research (NITER), Constituent Institute of the University of Dhaka, Savar, Dhaka 1350, Bangladesh; c Department of CSE, Green University of Bangladesh, 220/D, Begum Rokeya Sarani, Dhaka 1207, Bangladesh; d Future Technology Research Center, College of Future, National Yunlin University of Science and Technology, 123 University Road, Section 3, Douliou, Yunlin 64002, Taiwan; e Institute of Information Engineering, Automation and Mathematics, Slovak University of Technology in Bratislava, Bratislava, Slovakia; f John von Neumann Faculty of Informatics, Obuda University, 1034 Budapest, Hungary; g Center for Computational and Integrative Biology, Rutgers University, Camden, NJ 08102, USA; h Department of Computer Science, Rutgers University, Camden, NJ 08102, USA

**Keywords:** Brain tumor, Magnetic reasoning imaging, Computer-assisted diagnosis, Convolutional neural network, Data augmentation

## Abstract

Detection and Classification of a brain tumor is an important step to better understanding its mechanism. Magnetic Reasoning Imaging (MRI) is an experimental medical imaging technique that helps the radiologist find the tumor region. However, it is a time taking process and requires expertise to test the MRI images, manually. Nowadays, the advancement of Computer-assisted Diagnosis (CAD), machine learning, and deep learning in specific allow the radiologist to more reliably identify brain tumors. The traditional machine learning methods used to tackle this problem require a handcrafted feature for classification purposes. Whereas deep learning methods can be designed in a way to not require any handcrafted feature extraction while achieving accurate classification results. This paper proposes two deep learning models to identify both binary (normal and abnormal) and multiclass (meningioma, glioma, and pituitary) brain tumors. We use two publicly available datasets that include 3064 and 152 MRI images, respectively. To build our models, we first apply a 23-layers convolution neural network (CNN) to the first dataset since there is a large number of MRI images for the training purpose. However, when dealing with limited volumes of data, which is the case in the second dataset, our proposed “23-layers CNN” architecture faces overfitting problem. To address this issue, we use transfer learning and combine VGG16 architecture along with the reflection of our proposed “23 layers CNN” architecture. Finally, we compare our proposed models with those reported in the literature. Our experimental results indicate that our models achieve up to 97.8% and 100% classification accuracy for our employed datasets, respectively, exceeding all other state-of-the-art models. Our proposed models, employed datasets, and all the source codes are publicly available at: (https://github.com/saikat15010/Brain-Tumor-Detection).

## Graphical abstract

**Figure:**
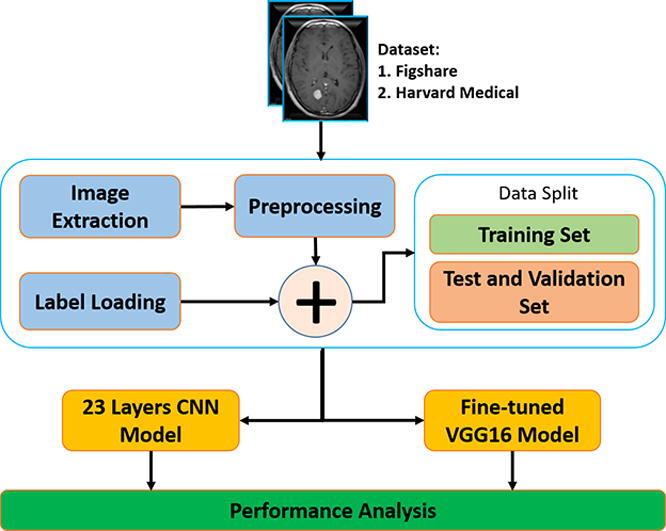

## 1 Introduction

A brain tumor is one of the deadliest illnesses which occurs due to the sudden and unregulated brain tissue growth inside the skull. It can be either benign or malignant. Malignant tumors can expand quickly and disperse across the surrounding brain tissue, whereas benign tumors tend to grow slowly. However, benign tumors can also be dangerous as their proliferation may affect surrounding brain tissues. About 70% of the tumors are benign, and 30% are malignant [1]. So far, more than 120 different brain tumors including meningioma, glioma, and pituitary as the most popular ones have been detected and identified. Among these three, meningioma tumors are perhaps the most prominent primary brain tumor in the meninges and affect the brain and spinal cord [2]. On the other hand, glioma tumors grow from glial cells called astrocytes. The most prominent tumor of glioma is an astrocytoma, a low-risk tumor that suggests slow development. However, high-risk glioma is one of the most severe brain tumors. Pituitary is another type of tumor that is due to excessive growth of brain cells in the pituitary gland of the brain. Therefore, early diagnosis of a brain tumor is essential due to its deadly aspect.

According to the International Association of Cancer Registries (IARC), there are more than 28,000 people diagnosed with brain tumors every year just in India in which more than 24,000 people die [3]. Another study reported that there are approximately 5,250 deaths recorded annually in the United Kingdom due to brain tumors [4]. In the United States, the impact of brain tumors is even more significant than in other countries. Just in 2019, about 86,970 cases of benign and malignant brain tumors are diagnosed [5]. The radiologist uses different experimental procedures for diagnosing brain tumors, including biopsy, Cerebrospinal fluid (CSF) analysis, and X-ray analysis. In the biopsy procedure, a small fragment of tissue is removed by surgery. The radiologist then determines whether the tissue holds a tumor or not. However, the biopsy process introduces many risks including inflammation and severe bleeding. It also has just 49.1% accuracy [6]. CSF is a colorless fluid that illustrates inside the brain. The radiologist tests the liquid to detect a brain tumor. However, similar to biopsy, it introduces many risks including bleeding from the incision site to the bloodstream and perhaps an allergic reaction after the treatment [7]. Similarly, using X-rays on the skull can lead to an increase in the risk of cancer due to the radiation.

Nowadays, image modalities are becoming more popular for radiologists since they are more accurate and introduce much less risk to patients. There are different methods for capturing medical imaging data including radiography, magnetic reasoning imaging (MRI), tomography, and echocardiography. Among them, MRI is the most prominent as it provides higher resolution images without any radiation. MRI is a non-invasive procedure that provides the radiologist with useful knowledge of medical image data to diagnose brain abnormalities [8], [9]. On the other hand, the Computer-Aided Diagnosis (CAD) method is designed for detecting brain tumors in the early stages without any human intervention. CAD systems can produce diagnostic reports based on MRI images and offer guidance to the radiologist [10].

The CAD process has improved dramatically using machine learning (ML) and deep learning (DL) applications in the medical imaging field [11], [12], [13]. Such techniques lead to better accuracy in terms of detecting brain tumors in the CAD system. Machine learning techniques are based on feature extraction, feature selection, and classification approaches. Different feature extraction techniques, including thresholding-based, clustering-based, contour-based, and texture-based are used for segmenting the tumor region from the human skull [14]. Such techniques extract the features from the MRI images where the important features are selected through the feature selection process. Extracting features with significant discriminatory information lead to achieving high accuracy [15]. However, using features extraction, it is possible to discard important information from the original image [16].

On the other hand, DL methods address this issue by using the original image as input[17]. In other words, they do not require handcrafted features for classification purposes. Among DL models, Convolutional Neural Network (CNN) provides[18] different convolution layers which will automatically extract features from the images[19]. CNN performed well when working with a large dataset which is not always easy to obtain in the medical imaging field [20]. One method to address this issue is to use transfer learning. In transfer learning[21], a model that has been previously trained with another large dataset related to another domain is used for the classification purpose[22]. Such knowledge helps the model to achieve high accuracy on a small dataset [23].

In this paper, we propose a system for automatically classifying brain tumors based on two deep learning models. A “Fine-tuned proposed model with the attachment of the transfer learning based VGG16” architecture is used for classifying normal and abnormal brain images. Four dense layers are employed in place of the completely connected layers during the tuning process, with the last dense layer equipped with a softmax activation function being used to identify brain tumors. To transform the two-dimensional matrix into a vector, we use Global Average Pooling 2D instead of flattening layers. A total of 71 normal and 81 abnormal MRI images are used in this classification to address the data imbalance problem. On the other hand, we propose a “23-layers CNN” architecture for classifying multiclass brain tumors. In this work, a total of 3064 MRI images are used for training the CNN model. A dropout layer is applied to solve the overfitting issue. In addition, different kernel sizes are integrated with the model to extract the complex features from the MRI images, making the model more robust. Our experimental results indicate that our models reach up to 97.8% and 100% prediction accuracies for our employed, exceeding all other previous studies found in the literature.

To summarize, the main contributions of this study are as follows:

• The “23-layer CNN” framework provides segmentation-free feature extraction techniques that do not require any handcrafted feature extraction method relative to the conventional machine learning methods.
• In this model, we replace the fully connected layers with four dense layers which facilitate the tuning process.
• Data imbalance issue is solved in the Harvard Medical dataset by taking an almost equal number of MRI slices in both normal and abnormal tumor classes.
• The overfitting issue is solved in this study by increasing the number of MRI slices using a data augmentation strategy and introducing the dropout layers within both models.
• The proposed “23-layers CNN” framework performance is evaluated on both large and small datasets. Results indicate that our framework is able to outperform previous studies found in the literature.
• To prevent overfitting in a small image dataset, we merged the “23-layers CNN” framework with the transfer learning-based VGG16 model. Results show that the suggested technique performs splendidly in the test images without experiencing any overfitting problems.

Our proposed models, employed datasets, and all the source codes are publicly available at:  https://github.com/saikat15010/Brain-Tumor-Detection.

## 2 Background

During the past decades, a wide range of machine learning and deep learning models for detecting brain tumors have been proposed. In this section, a summary of such models is presented.

### 2.1 Brain tumor detection with segmentation based machine learning technique

As a large volume of medical MRI imaging data is gathered through image acquisition, the researchers are now proposing different machine learning methods to identify brain tumors. These methods are based on feature extraction, feature selection, dimensionality reduction, and classification techniques. Most of those suggested machine learning models are focused on the binary identification of brain tumors. For example, Kharrat et al. proposed a binary classification of brain images using a support vector machine (SVM) and a genetic algorithm (GA) [24]. In this study, the features are extracted using Spatial Gray Level Dependency (SGLDM) method. In a different study, Bahadure et al., used Berkeley wavelet transformation (BWT) and SVM to segment and categorized normal and abnormal brain tissues [25]. They were able to achieve 96.5% prediction accuracy on 135 images. In a related study, Rehman et al., used a Random Forest (RF) classifier to the 2012 BRATS dataset [26]. They compared their model to other classifiers and found that the RF classifier achieve better results in terms of precision and specificity.

Later, for the purpose of identifying brain tumors, Chaplot et al. used a discrete wavelet transform (DWT) as a feature extractor and SVM as a classifier [27]. On 52 images, they achieved 98% prediction accuracy. The K-nearest neighbor (KNN) classifier was then applied by El-Dahshan et al. to 70 images, and the results showed 98.6% prediction accuracy [28]. For feature extraction and feature reduction, they employed DWT and the principle component analysis (PCA), respectively. They also used Particle Swarm Optimization (PSO) and SVM to select and classify textural features. To detect different grading of glioma tumors, Chen et al., used a 3D convolution network to segment the tumor region [29]. The segmented tumors are then classified using the SVM classifier. They also used the recursive function exclusion (RFE) method to extract features with significant discriminatory information. More recently, Ranjan et al., proposed a new model using 2D Stationary Wavelet Transform (SWT) as a feature extractor, and AdaBoost and SVM classifiers to detect brain abnormalities.

Although those techniques significantly enhanced brain tumor detection accuracy, they still have several limitations, including:

• Since all these methods are based on binary classification (normal and abnormal), it is not sufficient for the radiologist to decide the patient’s treatment concerning tumor grading.
• Those methods are based on different hand-crafted feature extraction techniques, which are time-consuming, complex, and in many cases not effective.
• Techniques that were used in those studies performed well with a small amount of data. However, working with a large volume of data required advanced classifiers.

### 2.2 Brain tumor detection using convolution neural networks (CNN)

CNN presents a segmentation-free method that eliminates the need for hand-crafted feature extractor techniques. For this reason, different CNN architectures have been proposed by several researchers. Most of the CNN models reported multiclass brain tumor detection, including a vast number of image data. For example, Sultan et al., suggested a CNN model with 16 layers [30]. The CNN model tested on two publicly available datasets. One dataset identified tumors as meningioma, glioma, and pituitary tumors, and the other dataset differentiated between the three grades of glioma tumors, including Grade II, Grade III, and Grade IV. They achieved 96.1% and 98.7% prediction accuracies on datasets with 3064 and 516 images, respectively. Hossain et al., used the Fuzzy C-Means clustering technique to extract the tumor area from the MRI images [31]. They proposed a new CNN-basedmodel and compared it to six other machine learning models. The reported 97.9% prediction accuracy outperforms prior models.

A novel hybrid CNN model was created by Ertosun et al. in a different study to find multiclass glioma tumors [32]. For Grade II, Grade III, and Grade IV glioma tumors, they achieved classification accuracy of 96.0%, 71.0%, and 71.0%, respectively. In a similar study, Anaraki et al., identified glioma tumors with 90.9% prediction accuracy using CNN and GA [33]. They obtained 94.2% prediction accuracy for the diagnosis of pituitary, meningioma, and glioma tumors. More recently, Özyurt et al., suggested a combined Neutrosophy and CNN model. In this model, the Neutrosophy technique is used to segment the tumor zone, the segmented portion is extracted using the CNN model and then classified using SVM and KNN classifiers [34]. In a different study, Iqbal et al., introduced a 10-layer CNN model to tackle this problem [35]. They carried out their experiment on the BRATS 2015 dataset and achieved promising results. As it is discussed here, CNN appears to be doing well for a large image dataset. However, it also suffers from two main limitations as follows:

• CNN model required a vast number of images for training, which is often difficult to obtain in the medical imaging field.
• Convolutional Neural Networks (CNN) perform remarkably well at classifying images that are quite similar to the dataset. CNNs, on the other hand, struggle to classify images that have a slight tilt or rotation. This can be fixed by utilizing data augmentation to continuously introduce new variants to the image during training. To address this problem in our research, we employed the data augmentation technique.

### 2.3 Brain tumor detection through transfer learning

Transfer learning does well when the volume of data is limited since such a model is previously trained on a large dataset (e.g., the ImageNet database), containing millions of images. In this approach, the pre-trained model with adjusted weights is adopted for the classification tasks. Another benefit is that it does not require a massive amount of computational resources since only the model’s fully connected layers need to be trained. Due to such advantages, different transfer learning models have been used for diagnosing brain tumors. For instance, Talo et al., used a pre-trained ResNet34 model to detect normal and abnormal brain MRI images. A large-scale of data augmentation is also carried out to reach high prediction accuracy [36]. Furthermore, for detecting multiclass brain tumors, Swati et al., proposed a fine-tuned VGG19 model [37]. Later on Lu et al., suggested a fine-tuned AlexNet structure to diagnose brain abnormalities [38]. In this study, just 291 images were used. In a similar study, Sajjad et al., used a fine-tuned VGG19 model for multiclass brain tumor detection and conducted it on 121 images [39]. They achieved an overall prediction accuracy of 87.4% before the data augmentation. Finally, by applying the data augmentation technique, they increased the accuracy to 90.7%. Despite all the benefits, there are several shortcomings associated with transfer learning which are listed below:

• Pre-trained models fail to obtain satisfactory results when training on imbalance datasets. They are more biased towards classes with a larger number of samples [36] [38] [56].
• Proper fine-tuning is required in pre-trained models. Otherwise, the model will fail to achieve satisfactory results [37] [39].

Although previous studies achieved significant improvement in brain tumor diagnosis, there is still room for improvement. This research mainly concentrated on overcoming those shortcomings by fine-tuning the deep learning models and improving forecast accuracy.

## 3 Methodology

**Fig. 1:**
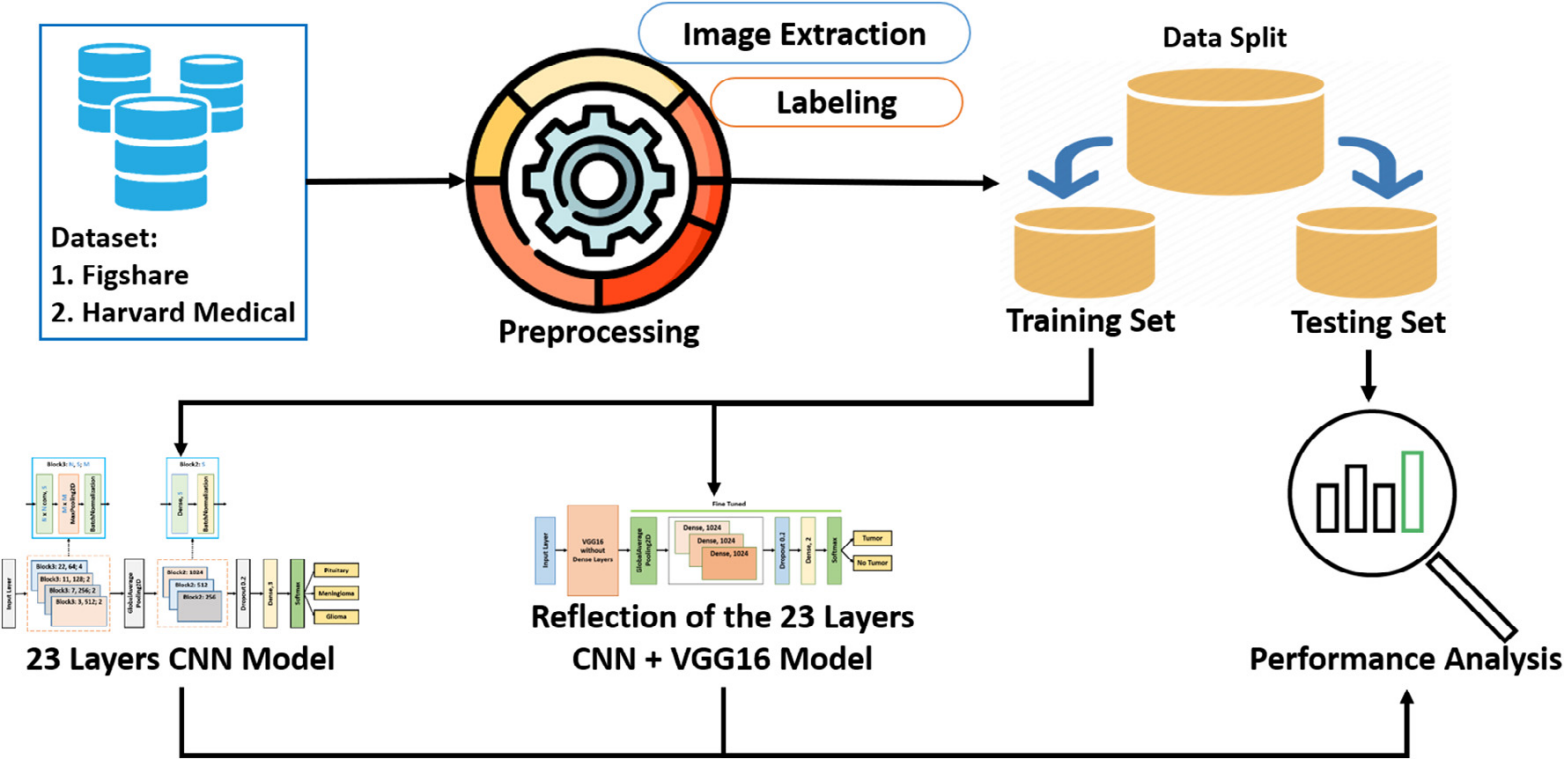
Proposed architecture for brain tumor detection..

Our proposed block diagram for automated binary and multiclass brain tumor detection is shown in Fig. 1. The architecture starts with image extraction and loading labels from the dataset. The extracted images then need to be preprocessed before splitting them into training, validation, and test set. Finally, our proposed “23-layers CNN” and the “Fine-tuned VGG16” architectures are applied to the employed datasets. In the following sections, the block descriptions of our proposed methods are discussed in detail.

### 3.1 Dataset

**Fig. 2:**
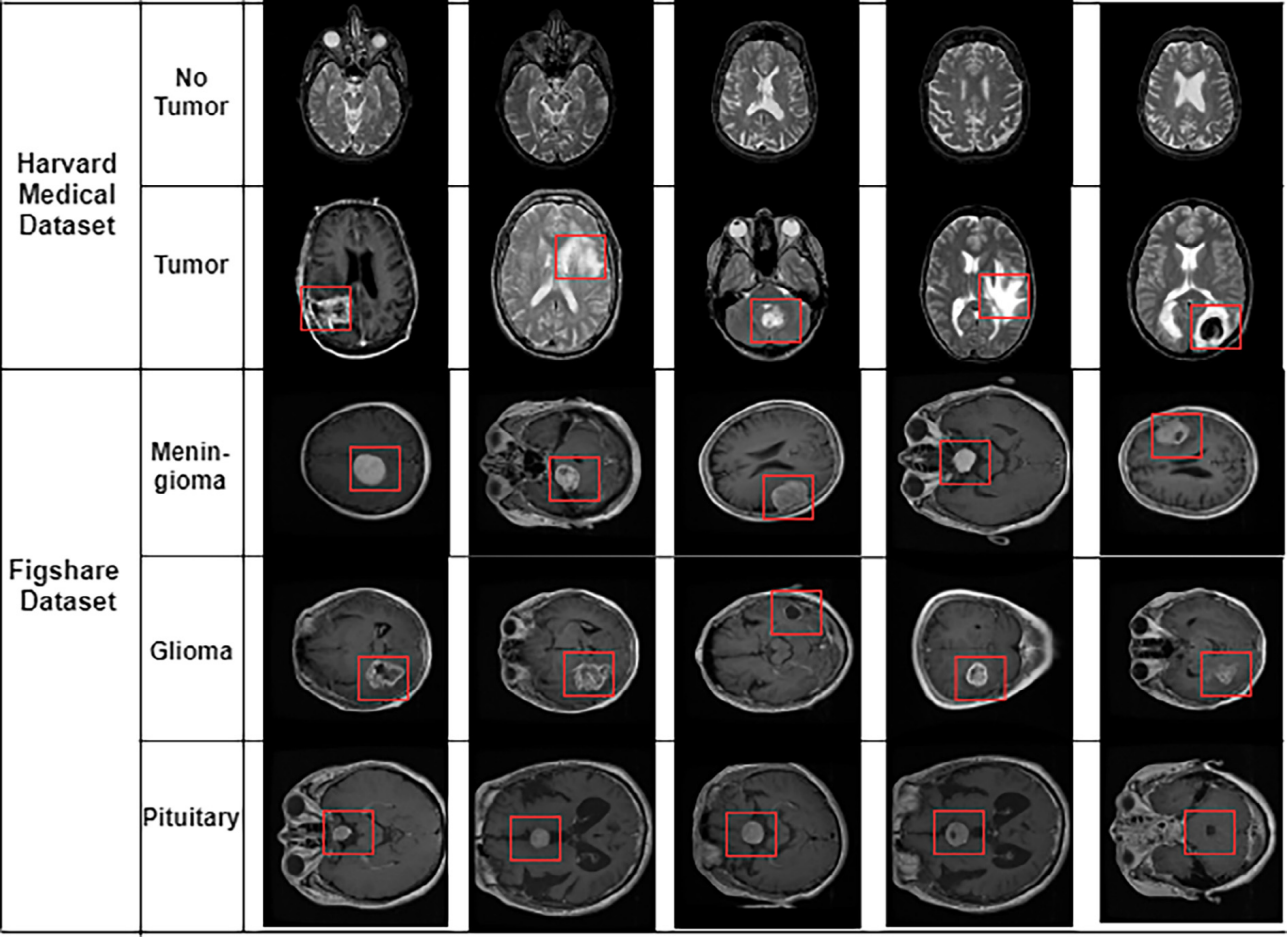
Different samples of brain tumors. Glioma, Metastatic adenocarcinoma, Metastatic bronchogenic carcinoma, Meningioma, and Sarcoma tumors from left to right in Harvard medical dataset. The tumor presents within the rectangle.

In this study, two different datasets are used. The first one (referred to as dataset 1 in this article) is a publicly available CE-MRI Figshare dataset [40]. The data was collected from General Hospital, Tianjin Medical University, and Nanfang Hospital (China) during 2005 to 2010. This dataset contains a total of 3064 T1- weighted contrast MRI slices from 233 patients diagnosed with one of the three brain tumors, including meningioma, glioma, and pituitary (as shown in Fig. 2). The MRI images used in this dataset have three different views including axial, coronal, and sagittal.

The second dataset (referred to as dataset 2 in this article) is collected by the Harvard repository [41]. The dataset includes a total of 152 T1 and T2-weighted contrast MRI slices. Among them, 71 slices are healthy images that do not contain any tumor, and a total of 81 are abnormal images containing a tumor. The abnormal brain slices have five different types of tumors, including Glioma, Metastatic adenocarcinoma, Metastatic bronchogenic carcinoma, Meningioma, and Sarcoma (as shown in Fig. 2). Table 2, Table 1 include detail information of these two datasets.

**Table 1:** Number of MRI slices in dataset 2.

Brain	Tumor Class	Number of Slices
Normal	Normal Image	71
Abnormal	Glioma	29
	Metastatic audenocarcinoma	8
	Metastatic bronchogenic carcinoma	12
	Meningioma	16
	Sarcoma	16
Total		152

**Table 2:** Number of MRI slices in dataset 1.

Tumor Class	Number of Patients	Number of MR Slices
Meningioma	82	708
Glioma	91	1426
Pituitary	60	930
Total	233	3064

### 3.2 Data preprocessing

We employ several preprocessing techniques before feeding the images into our classifiers. For instance, all the MRI images in the Figshare dataset are in.mat type (defined in Matlab). Hence, to read the image, we require to expand the dimension of the image. After that, we transform all the images into NumPy arrays (available in python) so that our model can take up less space. Before splitting the dataset, we have shuffled the data so that our model can train on unordered data. After shuffling the data, we divide the dataset into three sections including train, test, and validation. Approximately 70% of the data is used for training, and a further 30% is used for validation and testing purposes (see Table 4).

**Table 4:** MRI slices distribution for training validation and testing purposes.

Dataset	Brain Tumor Type	Training	Validation	Testing
Harvard Medical	Normal	357	42	14
	Abnormal	406	49	16
Figshare	Meningioma	502	56	150
	Glioma	1032	115	279
	Pituitary	674	75	181

On the other hand, all the MRI images in the Harvard Medical dataset are in.GIF type. To process the dataset, we have converted the MRI images to.JPEG type. To reduce the image’s dimensionality, we down-size the original image from 256 × 256 × 1 to 128 × 128 × 3. We replicate the pixel intensity value three times to create three channels according to the pre-trained VGG16 architecture input size. Although only 152 images are available in dataset 2, we have conducted several data augmentation techniques for solving the overfitting issue, increasing the dataset size, and making the model more robust [42], [49], [50]. Further descriptions of the data augmentation technique are provided in Table 3. As a result, the number of images increased from 152 to 884 after performing data augmentation. Additionally, we have used 70% of the data to train the model, and a further 30% of the data were used to validate and test the proposed method. (see Table 4).

**Table 3:** Data augmentation strategy used in this study.

Serial	Parameter	Value
1	shear range	0.2
2	zoom range	0.2
3	rotation	90
4	width shift range	0.1
5	height shift range	0.1
6	vertical_flip	True
7	horizontal_flip	True

### 3.3 Proposed 23-layers CNN architecture

**Fig. 3:**
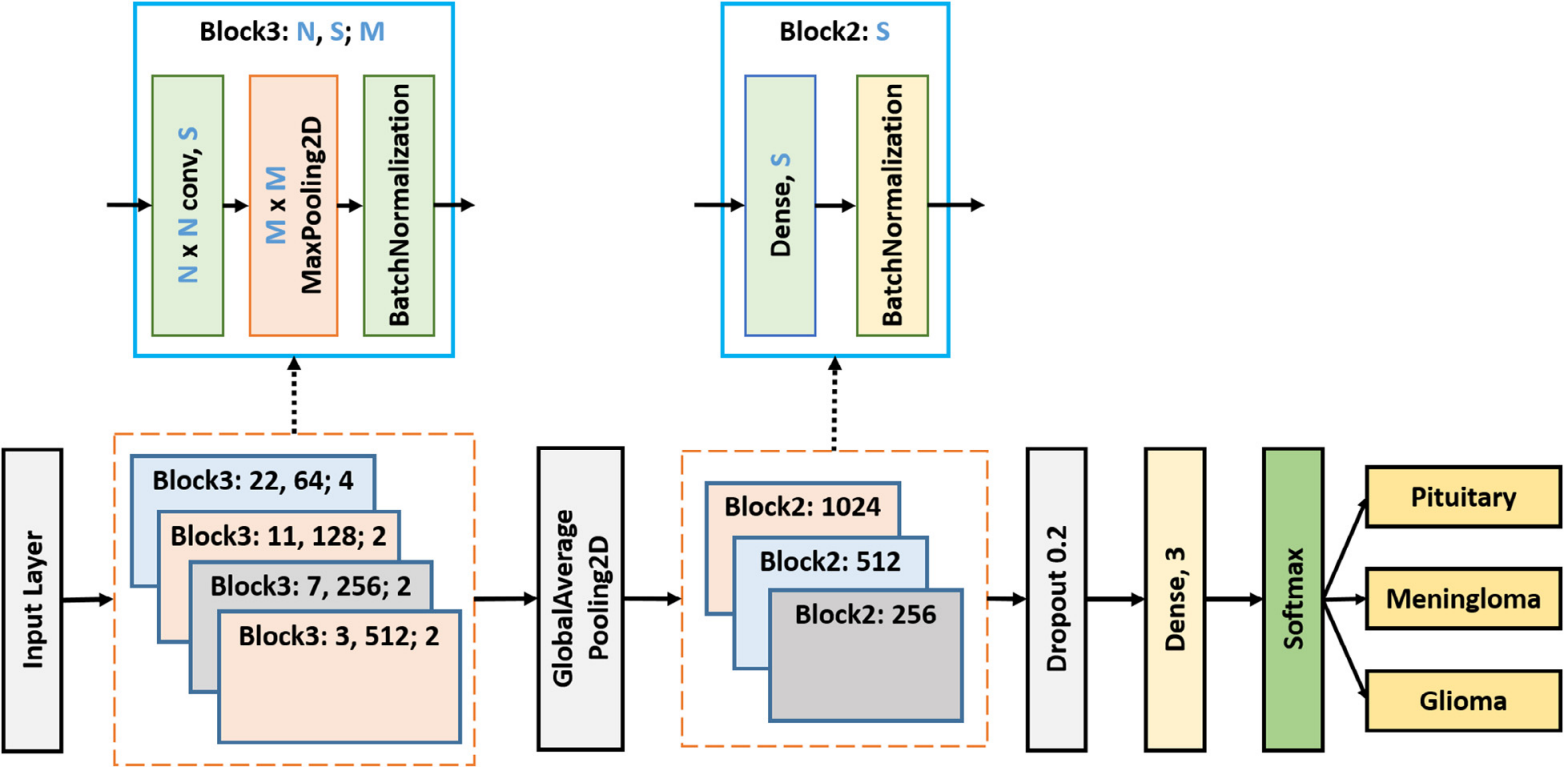
Proposed 23-layers CNN architecture.

**Fig. 4:**
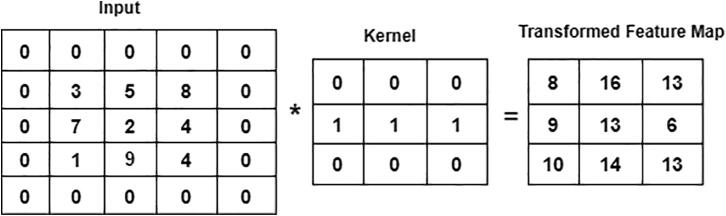
Convolution operation on 5 × 5 image using 3 × 3 kernel.

Fig. 3 demonstrates the proposed “23-layers CNN” architecture used to classify different tumor types, including meningioma, glioma, and pituitary. In the proposed architecture, we take MRI slices as input, process the slices in different layers, and differentiate them from one another. In this study, a total of 23 layers are used to process the slice. Below is the description of each layer:

One of the predominant building blocks of the CNN model is the convolutional layer. It is a mathematical method that performs a dot product between two matrices to construct a transformed feature map. One matrix relates to the kernel, while the other presents the pixel intensity values of the original image. The kernel is used to move vertically and horizontally over the original image to extract properties such as borders, corners, shapes, etc. When we move further into the model, it begins to find more better features like blurring, sharpening, texturing, and gradients direction [43]. A total of four convolutional layers with different kernel sizes, including 22 × 22, 11 × 11, 7 × 7, and 3 × 3, are included in the “23-layers CNN” architecture. We move the filter 2 pixels at a time using stride two over the input matrix. For padding, we preserve the original size of the image by applying zero paddings, to avoid losing the details of the image. The following equation describes the convolutional layer:

(1) $$C(h,d)=(k*f)(h,d)=\underset{i}{\sum }\underset{j}{\sum }k(h-i,d-j)f(i,j)$$

where, K is the image with a size of (h, d), and (i, j) corresponds to the kernel size value with an f-number of filters. Fig. 4 illustrates the convolutional approach to generate the feature map.

As an activation function, we use the Rectified Linear Unit (ReLU) which performs non-linear operations within the convolutional layer. The RelU activation function helps to solve the gradient vanishing problem using the backpropagation process [44]. The RelU is defined as follows:

(2) f(z)=max(0,z)

**Fig. 5:**
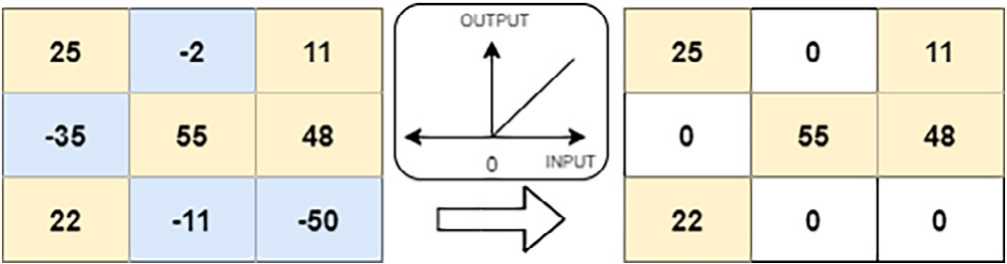
ReLU operation.

The ReLU activation function is graphically presented in Fig. 5.

In the next level, Pooling layers help to minimize the dimension of the transformed feature map. In this architecture, a total of 3 pooling layers are used. Different pooling layers are available in the CNN model, including max pooling, min pooling, and average pooling. We choose max pooling with varying sizes of the pool, such as 4 × 4 and 2 × 2, to retrieve the most prominent features from the transformed feature map [45]. Fig. 7 illustrates the max-pooling procedures where the feature map is in 4 × 4 blocks. As shown in this figure, max-pooling generates the most dominant features in every 2 × 2 blocks.

Batch normalization also plays a vital role in designing an accurate CNN model. It is used to regulate the model and enables a higher learning rate. It also helps to re-scale all the data to normalize the input data. Here we use a total of 7 batch normalization layers to build our model. Before feeding the data into a fully connected layer, GlobalAveragePooling2D is used to convert multi-dimensional data into a one-dimensional vector. It takes the average output of each convoluted feature map from the previous layer and build a one-dimensional vector. Next, the one-dimensional vector is fed into the fully connected layer as the input. Additionally, we employ a total of four fully connected layers to construct our model, with the classification taking place in the final fully connected layer. We have used softmax function as our activation function in the output layer of our proposed model, that predicts a multinomial probability where the probabilities of each value are proportional to the relative scale of each value in the vector. In the softmax activation function, the outcome value is between 0 and 1 which is defined as follows:

(3) $$\mathrm{softmax}{\left(z\right)}_{j}=\frac{\exp\left(z_{j}\right)}{\overset{n}{\underset{i=1}{\sum }}\exp\left(x_{i}\right)}$$

**Fig. 6:**
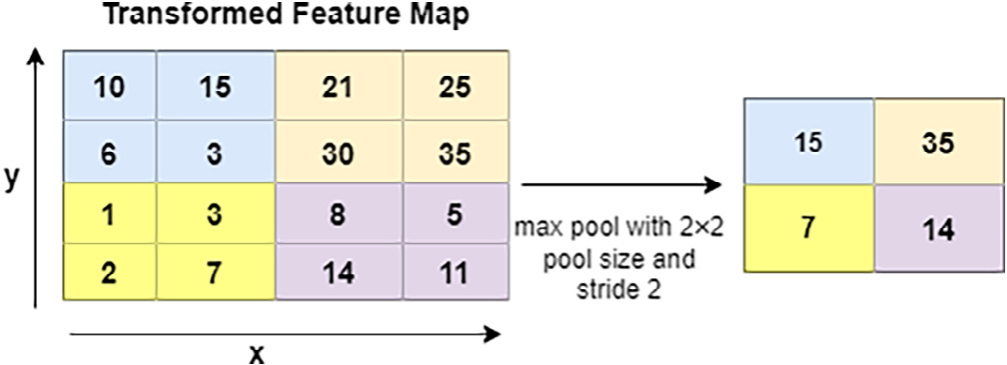
Dropout layer.

One of the most challenging issues in building an accurate deep neural network is overfitting. It occurs when the model is over-trained on the training data but has a negative impact on the new data [46]. To avoid overfitting, we use the dropout layer before the classification layer. In the “23-layers CNN” architecture, a dropout of 20% is used. Hence, only 80% of the features will be trained on every iteration. Fig. 6 illustrates the dropout procedure.

### 3.4 Fine-tuning for proposed CNN

A fine-tuning approach not only replaces the pre-trained model’s layers with a new set of layers to train a given dataset, it also uses backpropagation to fine-tune all or part of the kernels in the pre-trained convolutional layer. In this study, the Fine-tuned CNN pre-trained model is used to identify whether or not the tumor is located inside the image. As our pre-trained model, we use VGG16, which was first introduced in 2014 and became the first runner-up in the ILSVRC competition [47]. When a model fits the training set too well, then overfitting happens. The model thus has a hard time generalizing to new data that are not in the training set. In the case of dataset 2, since the training dataset is small, it is very likely to overfit complex models. To address this issue, we combine the reflection of our proposed “23-Layers CNN” architecture with the “transfer learning based VGG16 architecture”. The VGG16 architecture was fine-tuned to be integrated with the reflection of the proposed model with Harvard Medical dataset (as presented in Fig. 8).

**Fig. 7:**
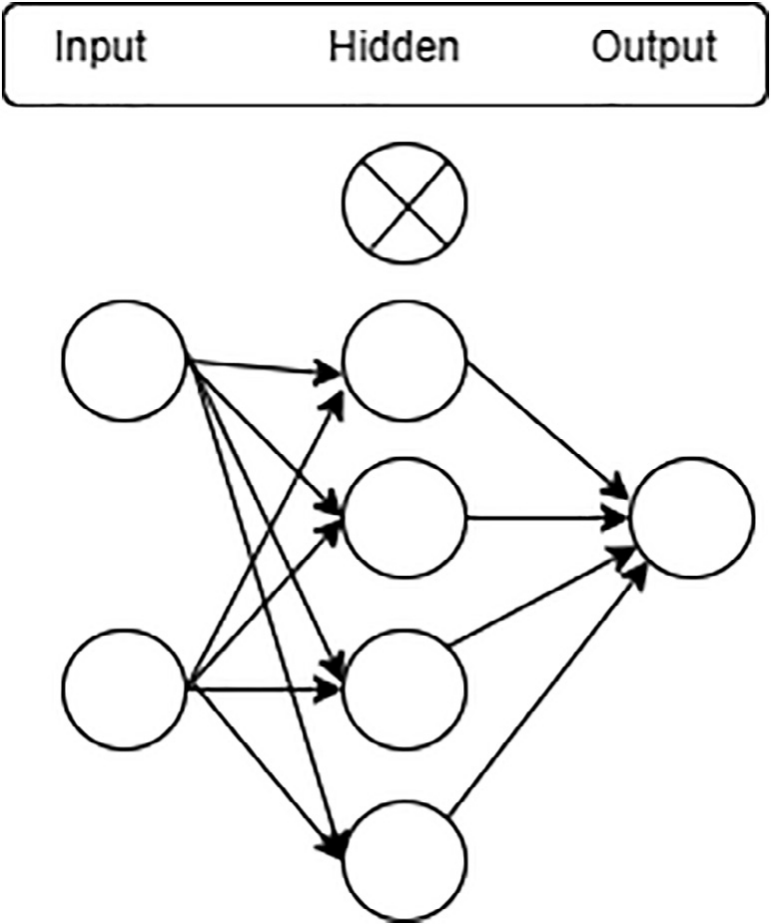
Max Pooling procedure.

**Fig. 8:**
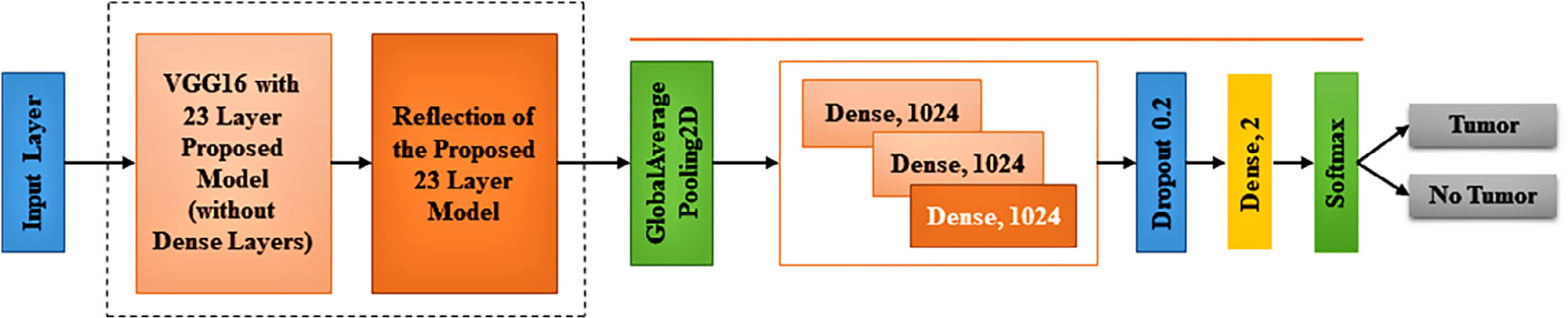
Fine-tuned Proposed architecture with the attachment of “transfer learning based VGG16 architecture”..

Here we use all 13 convolution layers from the VGG16 architecture along with the reflection of the proposed architecture with kernel size 3 * 3 and 5 total max-pooling layers with stride 2. In all convolution layers, the ReLU activation function is used. In this study, different filter sizes are used to fine-tune the fully connected layers, including 1024, 1024, 512, and 2. A dropout layer which is placed between two dense layers is also used for the fine-tuning process to overcome the over-fitting problem. Finally, in the classification stage, we use a CNN model and tune its parameters. We also investigate more about hyper-parameters such as padding, zero-padding, strides, feature map, batch size, and learning rate to build a best-suited model.

## 4 Experimental setup

The proposed models are implemented in TensorFlow, with Keras in Python. The implementation was performed on Google Colab which provides free online cloud service along with 15 GB of free space in google drive.

### 4.1 Training and parameter optimization

**Fig. 9:**
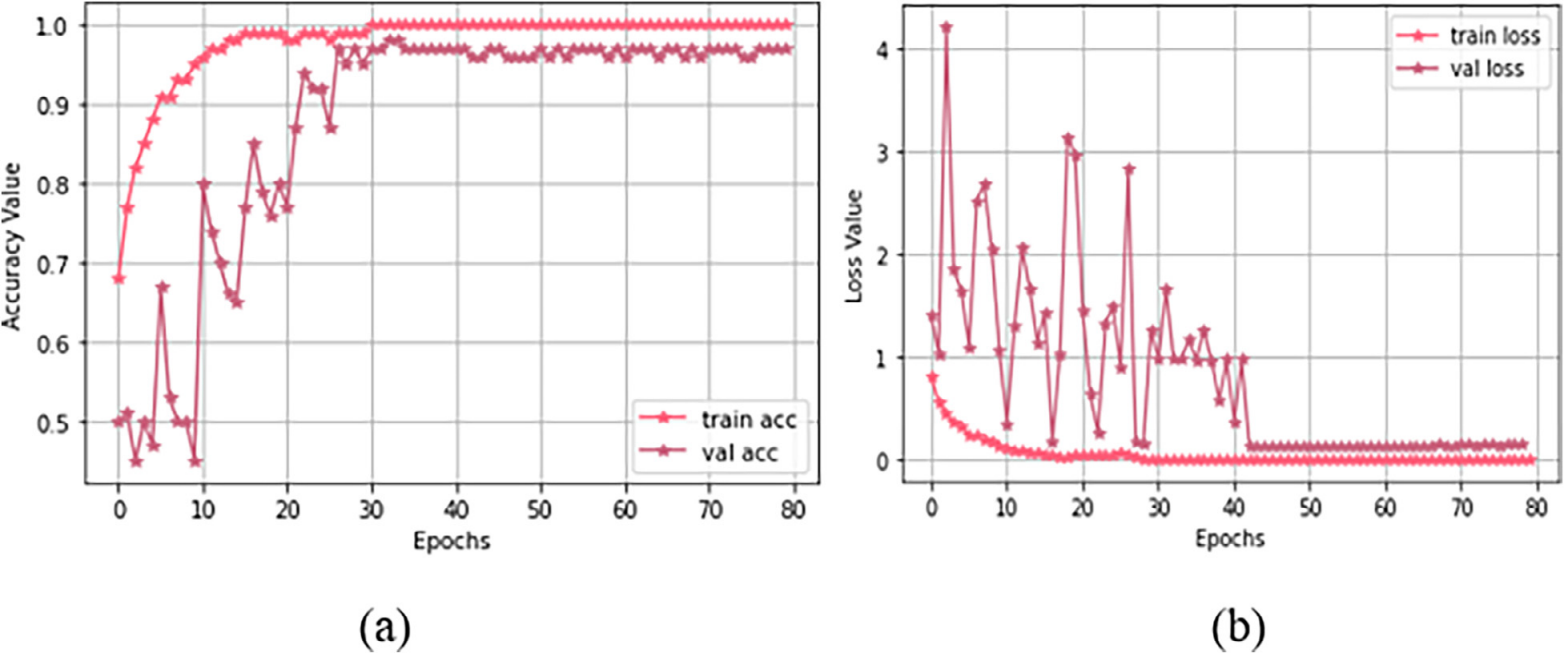
Training progress for study I: (a) accuracy value during training and validation process (preferred higher value), and (b) loss value during training and validation process (preferred lower value).

For Study I (using dataset 1), Fig. 9 demonstrates both training and validation steps for the “23-layers CNN” architecture. The hyper-parameter optimization used for this training is presented in Table 5. As our loss function, we select sparse categorical cross-entropy. We also study different batch-sized optimizers to train the model. Among them, the Adam optimizer with batch size 32 obtained the best performance. We observe that the optimal convergence for the model depends on the initial learning rate of alpha. We have to select alpha very carefully because CNN does not converge well if alpha is very high. If alpha is very small, then CNN will take more time to converge. Here we select the alpha as 0.0001 to avoid these issues.

**Table 5:** Optimization of Hyper-Parameters for Study I and Study II.

Model	Hyper-parameters	setting
CNN	Loss function	sparse_categorical _crossentropy
	Optimizer function	adam
	Metrics	accuracy
	Epochs	80
	Batch_size	32
	Learning_rate	0.0001
CNN- Pretrained Model	Loss function	categorical _crossentropy
	Optimizer function	adam
	Metrics	accuracy
	Epochs	40
	Batch_size	10
	Learning_rate	0.0001

For each epoch, Fig. 9(a) shows both training and validation progress. After the 29th epoch, the CNN model achieves 100% prediction accuracy with overall validation accuracy of 97.0%. Considering the consistency of the results (as shown in this figure), we can conclude that the “23-layers CNN” architecture successfully avoids the overfitting problem. Fig. 9(b) shows that the loss value decreases, and right after the 29th epoch, it hits zero for the training phase. Due to the limited batch size, some fluctuations occurred in the curve for the validation process. However the instability vanished after the 43rd epoch, and the loss curve approaches to zero.

## 5 Performance metrics

To evaluate the performance of “23-layers CNN” and “Fine-tuned VGG16” architectures and compare our results with previous studies, we use different evaluation metrics including, accuracy, precision, recall, false-positive rate (FPR), true negative rate (TNR), and F1-score. These metrics are calculate as follows:

(4) $$\mathrm{Accuracy}=\frac{\mathrm{TP}+\mathrm{TN}}{\mathrm{TP}+\mathrm{TN}+\mathrm{FP}+\mathrm{FN}}$$

(5) $$\mathrm{Precision}=\frac{\mathrm{TP}}{\mathrm{TP}+\mathrm{FP}}$$

(6) $$\mathrm{Recall}=\frac{\mathrm{TP}}{\mathrm{TP}+\mathrm{FN}}$$

(7) $$\mathrm{FPR}=\frac{\mathrm{FP}}{\mathrm{TN}+\mathrm{FP}}$$

(8) $$\mathrm{TNR}=\frac{\mathrm{TN}}{\mathrm{TN}+\mathrm{FP}}$$

(9) $$F1-\mathrm{score}=2\times \frac{\mathrm{recall}\times \mathrm{precision}}{\mathrm{recall}+\mathrm{prcision}}$$

Where TP stands for true positive, FP stands for false positive, TN stands for true negative, and FN stands for false negative.

## 6 Results

**Fig. 10:**
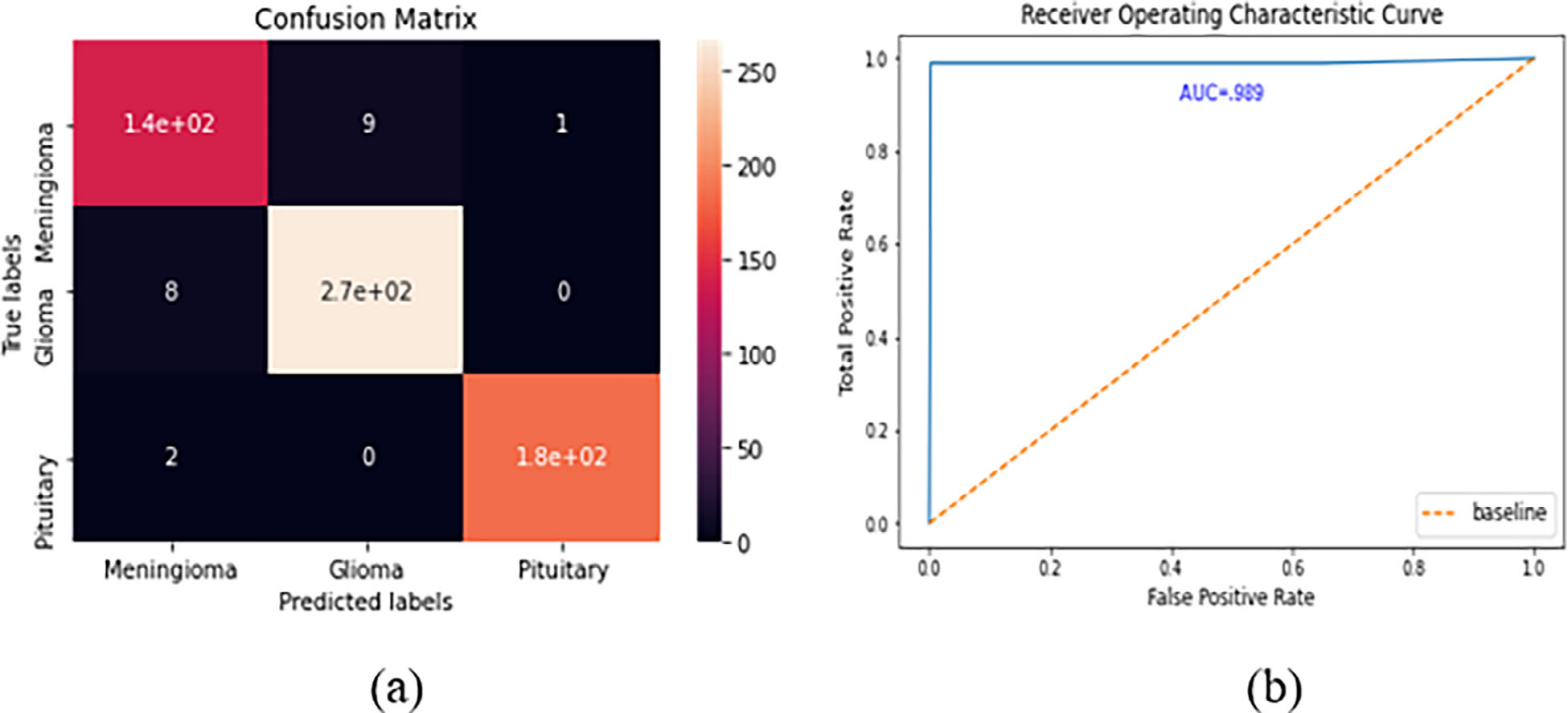
CNN model’s performance a) confusion matrix, b) ROC curve.

The confusion matrix and the ROC curve for the Figshare dataset are given in Fig. 10. In the Figshare dataset, a “23-layers CNN” architecture was used for the prediction purpose. It can be observed from Fig. 10 that a total of 140, 270, and 180 MRI slices are correctly classified for meningioma, glioma, and pituitary tumors, respectively. While only 20 MRI slices are misclassified by the proposed architecture. The other performance metrics, including accuracy, precision, recall, FPR, TNR, and F1-score, are presented in Table 6. As shown in Table 6, the prediction accuracy of 96.7%, 97.2%, and 99.5% are achieved for meningioma, glioma, and pituitary tumors, respectively. Finally, the overall prediction accuracy achieved on the Figshare dataset is 97.8%. For the other performance metrics, we achieve an average precision of 96.5%, a recall of 96.4%, and an F1-score of 96.4%. The false-positive rate is approximately 0, and the true negative rate appears to be close to 1, which demonstrates that the “23-layers CNN” architecture can achieve excellent efficiency on the Figshare dataset.

**Table 6:** The results obtained using the CNN model on dataset1.

Metrics	Tumor class	TP	TN	FP	FN	Accuracy	Precision	Recall	FPR	TNR	F1-score
Figshare Dataset	Meningioma	140	450	10	10	96.7%	93.3%	93.3%	0.021	0.978	0.933
	Glioma	270	323	9	8	97.2%	96.8%	97.1%	0.027	0.972	0.969
	Pituitary	180	427	1	2	99.5%	99.4%	98.9%	0.002	0.998	0.991
	Average Score					97.8%	96.5%	96.4%	0.016	0.983	0.964

From the ROC curve, we can observe that the area value is 0.989, which indicates the consistency and generality of our model.

### 6.1 System validation

We also apply our proposed “23 layers CNN” architecture to the Harvard Medical dataset. Here we achieved more than 85% training and validation accuracy on this dataset. However, the testing accuracy is less than 55%, indicating an overfitting issue occurred while training the model. Hence, to validate the system’s performance and for solving the overfitting issue, the generalization technique was applied. As it was discussed earlier, to build this model, we combine VGG-16 model with some reflection of our proposed “23 layers CNN” architecture as shown in Fig. 8. In this way, we address the overfitting issue for the small dataset.

Fig. 14 demonstrates both training and validation process for the “Fine-tuned VGG16” architecture. The hyper-parameter optimization used for the training process is presented in Table 5. At first, we have selected a minimal batch size of 10 since dataset 1 consists of only 152 MRI images. Additionally, we used categorical cross-entropy as a loss function, which is used in both single label and multi-class classification problems. We can observe from Fig. 14(a) that, right after the 33rd epoch, 100% training accuracy is achieved. As shown in Fig. 14(b), the loss value starts decreasing and after the 33rd epoch, it approaches to zero for both training and validation sets.

**Fig. 11:**
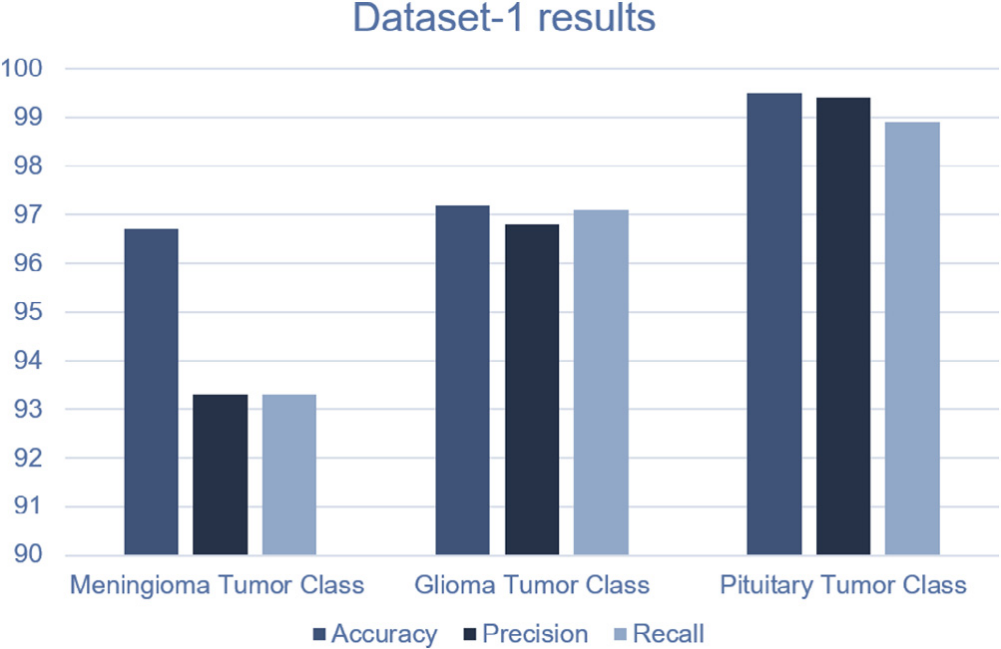
Performance of the proposed method on Dataset-1.

**Fig. 12:**
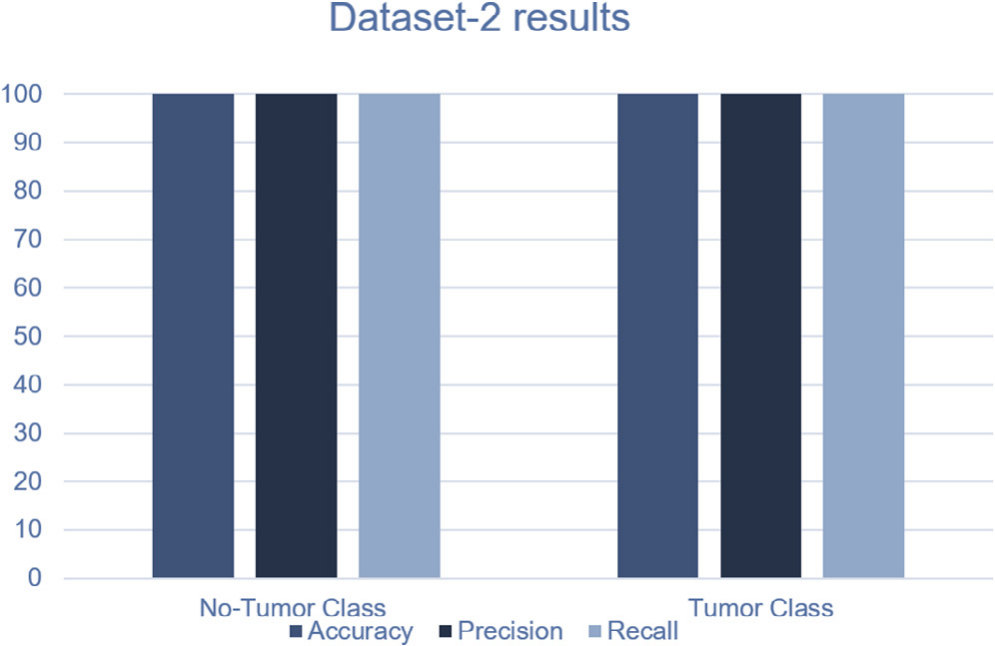
Performance of the proposed method on Dataset-2.

**Fig. 13:**
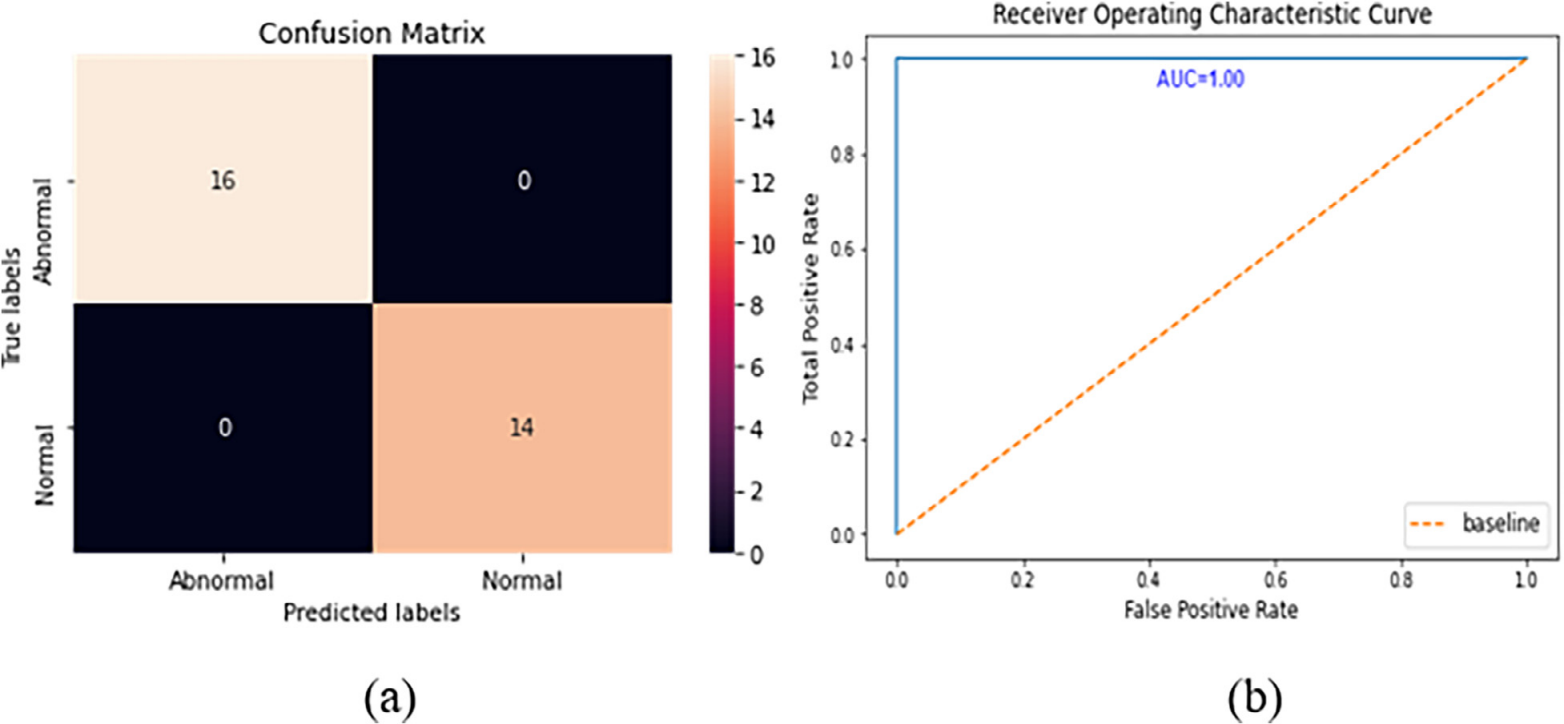
Fine-tuned model’s performance a) confusion matrix, b) ROC curve.

The confusion matrix and the ROC curves for dataset 1 are given in Fig. 13. In this dataset, a “Fine-tuned VGG16” architecture is tested on 30 images. Among them, 14 images contain no tumor, and 16 images include tumors. Interestingly, no MRI slices are misclassified by our proposed architecture. As shown in Fig. 13 all 14 and 16 MRI slices are correctly classified for normal and abnormal brain images, respectively. The other performance metrics are shown in Table 7. As shown in this table, we achieve an average accuracy of 100%, 100% precision, recall of 100%, and F1-score of 100%. The FNR is 0, and the TNR is 1 for dataset 2. From the ROC curve, we can also observe that the area under the curve value is 1, which indicates the model’s consistency and generality. The performance of the proposed framework on both datasets are given in Fig. 11, Fig. 12. We have also tested our proposed method using different configurations. Table 9 shows the performance of various activation functions and loss functions when combined with the proposed 23-layers CNN architecture. Among the loss functions, sparse categorical cross entropy performed well compared to the other two loss functions. Binary cross entropy, however, performed poorly. It is understandable that binary cross entropy will perform poorly when categorizing multiclass brain tumor grades because it worked well for the binary class data. The categorical cross entropy produced notable outcomes by obtaining greater than 90% accuracy. However, its performance was still inadequate to that of categorical cross-entropy. Additionally, we have employed three activation functions in this study where the softmax activation function and the sparse categorical cross-entropy loss function achieved more than 97% accuracy, outperforming all the other configurations.

**Table 7:** The results obtained using the reflection of the proposed CNN model on dataset2.

Metrics	Tumor class	TP	TN	FP	FN	Accuracy	Precision	Recall	FPR	TNR	F1-score
Harvard Medical Dataset	No Tumor	14	16	0	0	100%	100%	100%	0	1	100%
	Tumor	16	14	0	0	100%	100%	100%	0	1	100%
	Average Score					100%	100%	100%	0	1	100%

**Fig. 14:**
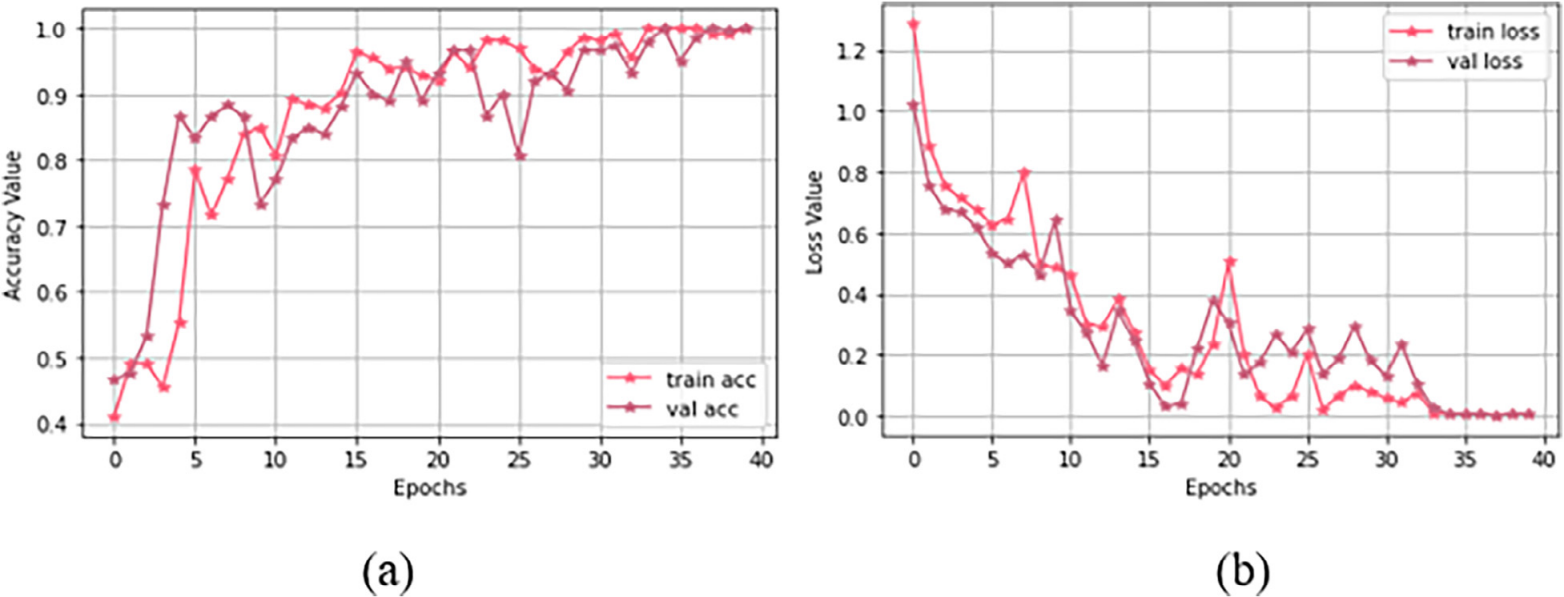
Training progress for study I I: (a) accuracy value during training and validation process (preferred higher value), and (b) loss value during training and validation process (preferred lower value).

## 7 Discussion

In this study, we proposed two individual models to diagnose binary (normal and abnormal) and multiclass (meningioma, glioma, and pituitary) brain tumors (see Fig. 1). The proposed models are compared to the existing state-of-the-art models found in the literature, which is illustrated in Table 8. Those models used the same datasets and tumor types with different architectures. It is evident from Table 8 that our proposed “23-layers CNN” and “Fine-tuned CNN with the attachment of transfer learning based VGG16” architectures demonstrate the best prediction performance for the identification of both binary and multiclass brain tumors compared to other methods found in the literature.

**Table 8:** Comparison of the proposed framework with the other state of art models

Method	Number of images	Classifier	Classification type	Accuracy
Shanaka et al. [52]	3064	Deep Learning + Active Contouring	Multi class	92
Momina et al. [53]	3064	Mask RCNN + ResNet-50	Multi class	95.9
Francisco et al. [54]	3064	CNN	Multi class	97
Emrah et al. [55]	3064	CNN	Multi class	92.6
Abiwinanda et al. [58]	700	CNN	Multi Class	84.1
Gudigar et al. [9]	612	PSO + SVM	Binary Class	97.4
El-Dahshan et al. [28]	70	KNN	Binary Class	98.6
Sultan et al. [30]	3064	CNN	Multi Class	96.1
Anaraki et al. [33]	3064	CNN + GA	Multi Class	94.2
Afshar et al. [48]	3064	CapsNets	Multi Class	90.8
Chaplot et al. [27]	52	SVM	Binary Class	98.0
Swati et al. [37]	3064	VGG19	Multi Class	94.8
Sajjad et al. [39]	3064	VGG19	Multi Class	94.5
Cheng et al. [51]	3064	SVM and KNN	Multi Class	91.2
Proposed Method	152	Fine-tuned VGG16	Binary Class	100
Proposed Method	3064	CNN	Multi Class	97.8

**Table 9:** Performance of different configurations on the Figshare dataset.

Method	Loss Function	Activation Function	Accuracy on Figshare Dataset
23-Layer CNN	Binary Cross Entropy	Sigmoid	82%
23-Layer CNN	Binary Cross Entropy	Tanh	80%
23-Layer CNN	Binary Cross Entropy	Softmax	84%
23-Layer CNN	Categorical Cross Entropy	Sigmoid	89%
23-Layer CNN	Categorical Cross Entropy	Tanh	91%
23-Layer CNN	Categorical Cross Entropy	Softmax	92%
23-Layer CNN	Sparse Categorical Cross Entropy	Sigmoid	94%
23-Layer CNN	Sparse Categorical Cross Entropy	Tanh	95%
23-Layer CNN	Sparse Categorical Cross Entropy	Softmax	97.8%

For the Harvard Medical Dataset (dataset 2) and Figshare dataset (dataset 1), we have obtained 100% and 97.8% prediction accuracies, respectively. However, there are other advantages to our proposed model over the existing models found in the literature. For example, most of the methods require handcrafted feature extractor methods [9] [27] [28]  [51], which may not be very effective when dealing with a large number of images. While the “23-layers CNN” and “Fine-tuned CNN with VGG16” architectures are segmentation-free and do not require handcrafted features.

Previously, Anaraki et al., introduced GA with CNN to predict brain tumors [33]. GA, however, does not always demonstrate good precision when working with CNN. GA is also a computationally expensive model. In another research, Afshar et al., used CapsNets architecture to focus on both the tumor and its surrounding region [48]. However, defining two objects at the same time can compromise the results for each individual problem despite their similarities. Swati and Sajjad et al., both applied the pre-trained VGG19 model to the Figshare dataset and obtained nearly the same performance [37] [39]. However, they did not implement any dropout or regularization strategy to solve the issue of overfitting.

In another study, Shanaka et al. segmented the tumor region using the active contour approach [52]. Active contour uses energy forces and limitations to extract the crucial pixels from an image for additional processing and interpretation. However, there are drawbacks that could occur while using active contouring in segmentation, such as getting stuck in local minima states while training or overlooking tiny details while minimizing the energy throughout the whole path of their contours. Momina et al. applied Mask RCNN along with the ResNet-50 model to locate the tumor region [53]. They have achieved 95% classification accuracy. However, more sophisticated object detection algorithms, such as the Yolo model and the Faster RCNN model, perform much better than the Mask RCNN. For instance, Eko et al. outperformed Mask RCNN by employing the Yolo model, which has a mAP rate of 80.12%, when segmenting the head and tail of fish [57].

Later on, Francisco et al., and Emrah et al. both used CNN model to obtain detection accuracy of more than 90% [54] [55]. However, both models are computationally expensive and do not offer a method for system validation. Since a specific model may work well on one dataset while having detrimental effects on another, it is crucial to apply system validation techniques. In a similar study, Abiwinanda et al. proposed a CNN model to categorize tumor classes using only 700 MRI images from the Figshare dataset [58]. They also did not employ any data augmentation techniques in order to increase the amount of MRI images. As a result, they only achieved a classification accuracy of 84%, which is quite low compared to similar studies.

**Fig. 15:**
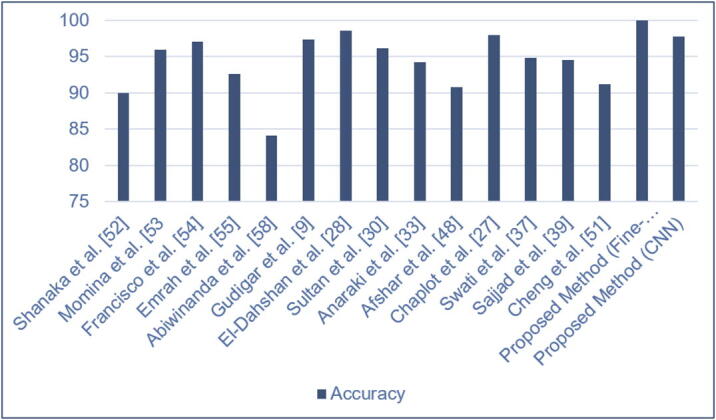
Performance of the proposed method compared to the latest research..

To classify the binary class, previous studies used an imbalance dataset [9] [27] [28]. We addressed this issue by using almost the same number of normal and abnormal brain MRI images. Besides, using the CNN model in the Figshare dataset, Sultan et al., achieved very promising results. However, there was still room for improvement by adding more layers into the network. A comparison between the proposed framework and all the previous studies found in the literature mentioned above are shown in Fig. 15.

### 7.1 Limitations and future work

Although our proposed models achieved promising classification outcomes, there are still a number of issues that can be resolved in the future work. For example, one of the key difficulties in using the deep learning-based automated detection of brain tumor is the requirement for a substantial amount of annotated images collected by a qualified physician or radiologist. In order to make a robust deep learning model, we would require a large dataset. To the best of our knowledge, the majority of contemporary machine learning tools for medical imaging have this constraint. Although the majority of earlier studies are currently making their datasets available to the public in an effort to address this problem. Sill, the amount of properly and accurately annotated data is still very limited.

Adopting zero-shot, few-shot, and deep reinforcement learning (DRL) techniques could help us to tackle this problem in the future. Zero-shot learning has the capacity to build a recognition model for unseen test samples that are not labeled for training. Zero-shot learning can thereby address the issue of the tumor classes’ lack of training data. Additionally, a deep learning model can learn information from a small number of labeled instances per class using few-shot learning technique. On the other hand, DRL can reduce the need for precise annotations and high-quality images.

Another drawback of this study is that although the proposed method achieved a significant performance on two publicly available datasets, the work is not validated on actual clinical study. It is the case for almost all of the models reviewed in this study as well. Our aim is to test our model on actual clinical data when thy become available. In this way, we can directly compare the performance of our proposed models with experimental approaches. Another future direction is to use more layers or other regularization techniques to work with a small image dataset using CNN model.

## 8 Conclusion

This research introduces two deep learning models for identifying brain abnormalities as well as classifying different tumor grades, including meningioma, glioma, and pituitary. The “proposed 23-layer CNN” architecture is designed to work with a relatively large volume of image data, whereas the “Fine-tuned CNN with VGG16” architecture is designed for a limited amount of image data. A comprehensive data augmentation technique is also conducted to enhance the “Fine-tuned CNN with VGG16” model’s performance. Our experimental results demonstrated that both models enhance the prediction performance of diagnosis of brain tumors. We achieved 97.8% and 100% prediction accuracy for dataset 1 and dataset 2, respectively outperforming previous studies found in the literature. Therefore, we believe that our proposed methods are outstanding candidates for brain tumor detection. Our proposed models, employed datasets, and all the source codes are publicly available at:  https://github.com/saikat15010/Brain-Tumor-Detection.

## Authors contributions

SIK, AR, and MKN conceived and initiated this study. SIK, AR, RK, and TD performed the experiments. SIK, AR, TD, SSB, AM, and ID wrote the manuscript. SIK, AR, MKN, SSB, MS, and ID helped with the literature review. AR, SSB, AM, ID, and TD mentored and analytically reviewed the paper. All the authors reviewed the article.

## Declaration of Competing Interest

The authors declare that they have no known competing financial interests or personal relationships that could have appeared to influence the work reported in this paper.
